# Optimal scale sizes and average-profit efficiency under uncertainty: A chance-constrained DEA approach

**DOI:** 10.1371/journal.pone.0295241

**Published:** 2024-06-27

**Authors:** Leila Parhizkar Miyandehi, Alireza Amirteimoori, Sohrab Kordrostami, Mansour Soufi

**Affiliations:** 1 Department of Applied Mathematics, Rasht Branch, Islamic Azad University, Rasht, Iran; 2 Department of Mathematics, Lahijan Branch, Islamic Azad University, Lahijan, Iran; 3 Department of Industrial Management, Rasht Branch, Islamic Azad University, Rasht, Iran; University of Tehran, ISLAMIC REPUBLIC OF IRAN

## Abstract

When the costs of the inputs and outputs of the units under evaluation are known, the evaluation of the profit efficiency of the units is one of the most significant evaluations that can provide valuable information about them. In this research, first, a new definition of the optimal scale size based on the maximization of the average measure of profit efficiency is presented. The average measure of profit efficiency develops the concept of economic efficiency measure by introducing a more accurate measure of efficiency compared to the measure of comparative and profit efficiency. It has been shown that the average measure of profit efficiency in a convex space is equivalent to the measure of profit efficiency in constant returns to scale technology, and then, some models are presented to calculate profit efficiency in a stochastic environment, to increase the ability of profit models in real examples by considering the calculation errors of inputs and outputs. Finally, the proposed method is used in an empirical example to calculate the average profit efficiency of a set of postal areas in Iran.

## Introduction

Data Envelopment Analysis (DEA), as a non-parametric approach, is a powerful tool for the evaluation of the performance of decision-making units (DMUs). It was presented by Charnes et al. [1] and then rapidly developed in various fields. Cost efficiency is one of the concepts that has attracted the attention of most researchers in the DEA literature. As long as, in addition to inputs and outputs, the costs of unit input values are available, the unit cost efficiency provides valuable economic information. In this regard, Cesaroni and Giovannola [2] first expressed the concept of optimal scale size (OSS) in the non-convex FDH technology. They introduced the OSS as one point in the production possibility space which minimizes the average cost efficiency (ACE). They showed that the average cost efficiency defines a measure of economic savings in efficiency analysis by combining two efficiencies of scale and allocation. This measure is a more accurate performance measure than cost and scale efficiencies. They also showed that ACE in a convex space with variable returns to scale is equivalent to cost efficiency with constant returns to scale, and therefore it is possible to calculate ACE in both convex and non-convex spaces without any assumptions about returns to scale.

Now, if we have the output price vectors of the units under evaluation besides the input cost vectors, the profit efficiency evaluation of the units can have more valuable information about the units compared to their cost efficiency. In this situation, the concepts of OSS and ACE will no longer be applicable because the basis for calculating them is only the cost of the inputs, and therefore, valuable income information is not included in these measures. Therefore, the development of the ACE measure to APE measure can provide a valuable and reliable evaluation of units while maintaining the features presented in ACE. Although there are studies on the profit efficiency of units in the DEA literature, none of them have addressed the concept of average profit efficiency (APE) and profit-based optimal scale size (OSSP). At the same time, to increase the ability to evaluate the profit performance of units, the concepts of average cost efficiency and optimal scale size addressed in the DEA literature can be developed for profit. Therefore, in this work, first, the concepts of average profit efficiency (APE) and optimal scale size are defined based on profit maximization. These concepts are an extension of Cesaroni and Giovannola [2] research on the profit of units under evaluation. The concept of OSSP is defined as a point in the production possibility set that maximizes the average radial profit for the unit under investigation. Based on this, the APE efficiency measure is an evaluation criteria of unit profit, which is far more accurate than the efficiency of profit and scale. In the following, methods of calculating APE in two convex and non-convex spaces are examined and it is shown that the APE model in convex space with variable returns to scale is equivalent to the profit model with constant returns to scale.

Considering that in the real world we are dealing with uncertain data, it seems mandatory to use the stochastic programming method in which inputs, outputs, or both are affected by economic factors, political factors, the impact of unexpected government decisions, currency fluctuations, environmental and agricultural factors are out of administrator’s control. In this sense, many researches have been conducted in the field of stochastic DEA. Nevertheless, none of the conducted researches have evaluated the profit efficiency of units with stochastic inputs and outputs. Therefore, in this work, after proving the equivalence of the profit efficiency evaluation model with the APE model in convex space, a model of profit efficiency in the stochastic state is presented. This model, which is a probabilistic model with chance constraints, is first transformed into deterministic and then linearized during a process. Therefore, our proposed model allows us to calculate the measure of APE and OSSP of units (in which input and output values are uncertain under various factors) using a linear model and provides valuable information on the units under evaluation. In the end, in a practical example, the proposed method is used to evaluate the average profit efficiency of a set of postal areas in Iran, and the results are interpreted and analyzed.

**Fig 1:**
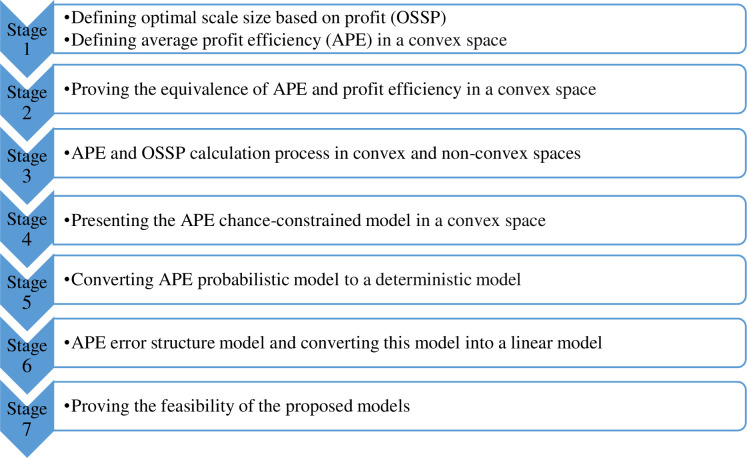
The research methodology.

The rest of this paper is structured as follows: In the Literature Review section, we review some of the most important researches in under study field. In the APE and OSSP section, the concepts of average profit efficiency and profit-based optimal scale size are defined, and models related to the calculation of average profit efficiency in convex and non-convex spaces are presented. Next, the concepts of average profit efficiency and optimal scale size are introduced in the stochastic environment and the relevant models are presented. Then the practical example is investigated by the proposed method, and finally, conclusions are drawn. The process can be briefly shown in the following flowchart (Fig 1):

## Literature review

Data envelopment analysis (DEA), initially propounded by Charnes et al. [1], is a prominent approach for evaluating the relative efficiency of decision-making units (DMUs). This non-parametric technique has rapidly been developed to analyze the performance in various conditions. One of these extensions is the inclusion of measurement errors and random data in evaluating DMUs. Land et al. [3] developed a chance-constrained DEA approach to calculate the efficiency of DMUs with deterministic inputs and random outputs with a normal distribution. Olsen and Peterson [4] considered a chance-constrained DEA model in multiplicative forms. Cooper et al. [5] considered joint chance constraints and presented an alternative chance-constrained DEA model. Ruggiero [6] addressed the measurement errors of frontier units as an average, in addition, to incorporate measurement errors in time periods. By considering the effect of measurement errors on the estimation of production frontier function, it was concluded that using models based on data average can lead to more realistic results. Khanjani Shiraz et al. [7] presented a joint chance-constrained DEA model to measure the performance of entities with crisp inputs and random outputs. Mehdizadeh et al. [8] have studied two-stage processes with random data. They introduced a stochastic network DEA approach based on the concepts of non-cooperative game theory and satisficing DEA to measure the efficiency of two-stage systems. Also, there are some studies such as Zhou et al. [9] and Amirteimoori et al. [10] in the DEA literature to analyze the performance of network systems with different structures and based on various assumptions while random performance measures are presented. Amirteimoori et al. [10] evaluated network systems in real applications using a multi-stage DEA model with probabilistic constraints. They evaluated the relative performance of supply chains and components in the presence of reverse flows and stochastic factors. Zhou et al. [9], considering data uncertainty, revised conventional leader-follower DEA models for two-stage systems. Then, they developed stochastic leader-follower DEA models to evaluate two-stage efficiency. Jahani Sayyad Noveiri et al. [11, 12] estimated sustainability performance and the most productive scale size using stochastic DEA from optimistic and pessimistic perspectives. Khanjani Shiraz et al. [13] assessed the economic efficiency (i.e. cost and revenue efficiencies) of DMUs in the presence of random inputs and outputs.

Farrell [14] as one of the pioneers of efficiency analysis showed cost efficiency as the whole efficiency, containing both technical efficiency and allocative efficiency. The cost efficiency of a DMU is described as its ability to reach the desired output level minimizing the total cost.

Frisch [15] was one of the first researchers to calculate the OSS that considered the OSS as a scale that maximizes the average physical productivity. DMUs with multiple inputs and outputs were considered in Frisch [15]. Then Baumol et al. [16] extended Frisch’s approach to investigate entities with several outputs. In the context of DEA, Färe and Grosskopf [17] defined the scale efficiency of the DMU under evaluation as the ratio of the cost efficiency of constant returns to scale (CRS) to the cost efficiency of variable returns to scale (VRS). Sueyoshi [18] also included the concept of economic scale size in the scale efficiency defined by Färe and Grosskopf [17]. Førsund and Hjalmarsson [19] stated that despite considerable efforts to classify and measure the OSS in DEA models, few studies have dealt with the best criterion for OSS in efficiency analysis. Cesaroni and Giovannola [2] first introduced the concept of OSS in the free disposal hull (FDH) non-convex technology set. They tried to find a point in the production possibility set to minimize the average cost efficiency (ACE). They also showed the ACE defines a size of economic savings in efficiency analysis combining two scale and allocation efficiencies that is a more accurate measure than cost and scale efficiencies individually. Moreover, it is demonstrated that ACE in convex space with VRS is equal to the cost efficiency value under the CRS assumption and therefore, ACE can be calculated in both convex and non-convex spaces without any assumption about returns to scale. Haghighatpisheh et al. [20] developed approaches to measure the average-cost efficiency, the average revenue efficiency, and average–cost/revenue efficiency, and also approximated the OSS by presenting a numerical heuristic method. Jahani Sayyad Noveiri et al. [12] dealt with OSSs and ARE of units under managerial disposability while there are undesirable outputs. Parhizkar Miyandehi et al. [21] extended Cesaroni and Giovannola’s research [2] to estimate the average revenue efficiency and OSSs in a stochastic environment. However, as we know, there is no DEA study to estimate the OSS based on maximizing profit under convex and non-convex technologies and also when random performance measures are presented. To illustrate, the extended model not only tackles revenue generation but also focuses on optimizing resources and reducing costs to increase profitability. By incorporating cost-effective strategies, streamlining operations, and maximizing resource allocation, the model aims to boost overall profitability. Furthermore, Parhizkar Miyandehi et al. [21] analyzed the performance of postal areas. In this current study, the efficacy of postal areas is also investigated while there are different in some areas. Simić et al. [22] presented a multi-phase functional model for the evaluation of the condition of road sections from the aspect of traffic safety. The main goal of their work was to develop a new multi-phase model consisting of CRITIC, FUCOM Fuzzy, DEA, and Fuzzy MARCOS to determine the level of traffic safety in parts of two-lane roads of the road network under conditions of uncertainty. Blagojević et al [23], based on the DEA method (CCR output-oriented model), presented a method to evaluate the efficiency of freight transportation railway companies. To solve the problem of criteria selection, they tested the FAHP hierarchical process method which showed the priority of evaluating the efficiency of railway companies based on 5 criteria groups. Akbarian et al. [24] have presented a new method for measuring the MPI of total profit. To calculate the total profit return intervals, cone ratio DEA models can be applied as weight constraints on the included information. Also, a new approach to calculate the upper limit of the overall profit yield of each DMU is presented.

In the study of Mitropoulos et al. [25], while pointing to the presence of noise in real-world data, a method based on stochastic DEA was used to measure performance, when endogenous (efficiency) and exogenous (patient satisfaction view) variables were inversely related. Agrawal et al. [26] evaluated the efficiency of 16 active private banks in India for the periods of 2015–2017 using DEA, Malmquist efficiency index, and SFA stochastic frontier analysis. The investigation shows evaluating the efficiency of Indian banks using DEA by the Malmquist productivity index and the stochastic frontier approach has been done for the first time in this study. Instead of evaluating and accompanying financial and sports data separately, Arsu [27] put both data in a single model. For efficiency analysis, BIO-MCDEA has been used which is a linear programming method for determining efficiency. Using Markowitz and cross-efficiency models for portfolio optimization and benefiting from the advantages of both models has not been considered enough in the literature. Rasoulzadeh et al. [28] combined a new hybrid Markowitz model and cross-efficiency model to introduce a four-objective model, which increased efficiency and reduced cross-efficiency covariance in addition to increasing returns and reducing portfolio risk. A non-dominant sorting genetic algorithm NSGA-II was also applied to solve the new model. Stević et al. [29] analyzed the efficiency of transportation companies by the integrated PCA-DEA model and multi-criteria decision-making methods. The main purpose of this study was to create an integrated model combining DEA, PCA (principal component analysis), CRITIC, entropy, and MARCOS methods for determining the efficiency of transportation companies in eight years.

Fathi [30] evaluated the super efficiency of water and sewage companies in the Markazi province of Iran by using stochastic DEA. Deterministic and stochastic super efficiency models have been investigated using the data from 2016 and the calculation results of both models have been obtained.

The ranking results of both models have been also evaluated for the water and sewage company. Kao et al. [31] developed a stochastic DEA model using the standard normal transformation, which can consider the correlation between the input and output factors of each production unit to find the stochastic efficiency distribution. Using the multiplicative model and the chance-constrained programming method, Piri et al. [32] evaluated the units with random data. Also, they developed its deterministic equivalent, which was a nonlinear program, and then proved that the deterministic equivalent of the multiplicative model can be converted into a quadratic programming problem. Gómez-Gallego et al. [33] evaluated the technical efficiency in the management of European health systems using DEA and FDEA models. They also evaluated the statistical relationship between the orientation in efficiency estimation and macroeconomic variables. The main results of this study showed a positive correlation between DEA and FDEA efficiency scores. Ngo et al. [34] combined SFA and DEA stochastic frontier analysis into a single framework to separate noise and inefficiency from DEA inefficiency scores. Accordingly, they provided confidence intervals for the estimated efficiency scores. Monte Carlo simulation showed that the presented model was a suitable alternative to conventional DEA and bootstrap DEA, and the validity of the new model was investigated in a practical example using data from Asia Pacific airlines between 2008 and 2015. Chowdhury et al. [35] evaluated the efficiency of Islamic banks in Southeast Asia with the aim of measuring the efficiency and productivity of Islamic banks in the SEA region. In this study, the DEA technique and productivity index Malmquist was used to evaluate the performance of 31 Islamic banks in SEA from 2014 to 2019. These studies were very effective for adopting effective policies in order to promote the ability and sustainability of SEA Islamic banks in the long term. Dar et al. [36] using three basic methods, namely DEA, Malmquist productivity index, and SFA showed how undesirable outputs such as NPA non-performing assets affect the technical efficiency of banks in India. Shah et al. [37] evaluated the efficiency and productivity growth of commercial banking industries in the South Asian region during the six years of 2008–2013. In addition, the technology gap between banking industries was investigated and the meta-frontier DEA technique was used to measure technical efficiency. Jahani Sayyad Noveiri and Kordrostami [38] assessed meta-frontier stochastic cost and revenue efficiencies of DMUs under the convex technology.

### APE and OSSP

Provided that output and input prices are available, the profit efficiency of systems can provide more valuable information than the cost efficiency or the revenue efficiency. In these circumstances, the ideas of ACE and OSS presented by Cesaroni and Giovannola [2] that are based on the cost of units will no longer be applicable. Therefore, extending the ACE to the APE can provide more useful insights while the features provided in the ACE are maintained. Consequently, we focus on estimating APE and OSSP in the following.

Suppose there are *n* DMUs, *DMU*_*j*_(*j*=1,2,…,*n*), which use *m* inputs *x*_*ij*_(*i* = 1, …,*m*) to produce *s* outputs *y*_*rj*_(*r* = 1, 2,…,*s*). The non-negative input and output vectors are also shown by *x*_*j*_ = (*x*_1*j*_,*x*_2*j*_,…,*x*_*mj*_)^*t*^and *y*_*j*_ = (*y*_1*j*_,*y*_2*j*_,…,*y*_*rj*_)^*t*^in which the superscript *t* shows the transpose of vectors and matrices. Considering the matrix of inputs and outputs as *X* = [*x*_1_,*x*_2_,…,*x*_*n*_]_*m*×*n*_ and *Y* = [*y*_1_,*y*_2_,…,*y*_*n*_]_*s*×*n*_, the FDH production possibility set under the VRS assumption is defined as follows:

$$T^{FDH}=\{(x,y)|X\lambda \leq x,Y\lambda \geq y,\sum_{j}\lambda_{j}=1,\lambda_{j}\in \{0,1\},j\in J\}$$ (1)

where *λ* is the *n*×1 vector with components equal to *λ*_*j*_, *j*∈*J* = {1,2,…,*n*}.

By incorporating the principle of convexity into the non-convex FDH production possibility set, the following technology proposed by Banker et al. [39] is made that the corresponding model has been called the BCC (Banker, Charnes, and Cooper) model.


$$T^{VRS}=\{(x,y)|X\lambda \leq x,Y\lambda \geq y,\sum \lambda_{j}=1,\lambda_{j}\geq 0,j\in J\}$$ (2)

The prices of inputs and outputs are shown by *P* = (*p*_1_,….,*p*_*m*_)>0 and *Q* = (*q*_1_,….*q*_*s*_)>0, respectively. Therefore, *Px*_*j*_ is the total cost which *DMU*_*j*_ spends to produce *y*_*j*_ and *Qy*_*j*_ is the total revenue *DMU*_*j*_ as the result of using *x*_*j*_. The values of input and output prices of all units are deemed to be the same. This assumption is only to simplify the notation, and the results can be generalized to cases that the input and output prices of the units are different. The concept of APE can be defined as the ratio of the average radial profit to the profit of the unit under evaluation. For more details, suppose the ray average profit of *DMU*_*j*_ (*RAP*(*x*_*j*_,*y*_*j*_)) is as follows:

$$RAP(x_{j},y_{j})=\frac{P(tx_{j},ty_{j})}{t}.$$ (3)

In Eq (3), *P*(*x*_*j*_,*y*_*j*_) is the total profit earned from the production plan (*x*_*j*_,*y*_*j*_), and *t*≠1 is a positive scalar that causes simultaneous expansion or contraction in the combination of inputs and outputs. By dividing statement (3) into the total profit of the unit *j*, we have:

$$\frac{RAP(x_{j},y_{j})}{P(x_{j},y_{j})}=\frac{P(tx_{j},ty_{j})}{P(x_{j},y_{j})}\times \frac{1}{t}.$$ (4)

Then consider (*x*_*h*_,*y*_*h*_)∈*T* as an arbitrary reference unit and determine the following:

$$\gamma_{j,h}=\underset{r}{\max}\{\frac{y_{rj}}{y_{rh}}\}\;\gamma_{j,h}\in (0,\infty ),$$ (5)

$$\rho_{j,h}=\underset{i}{\min}\{\frac{x_{ij}}{x_{ih}}\}\;\rho_{j,h}\in (0,\infty ).$$ (6)

By choosing $\gamma_{j,h}\leq k=\frac{1}{t}\leq \rho_{j,h}$, we can point out that $ty_{j}\leq \frac{1}{\gamma_{j,h}}y_{j}=\underset{r}{\min}\{\frac{y_{rh}}{y_{rj}}\}y_{j}\leq y_{h}$ and $tx_{j}\geq \frac{1}{\rho_{j,h}}x_{j}=\underset{i}{\max}\{\frac{x_{ih}}{x_{ij}}\}x_{j}\geq x_{h}$, *p*(*tx*_*j*_,*ty*_*j*_) = *p*(*x*_*h*_,*y*_*h*_) because of the strong disposability assumption of outputs. So, the last expression of the APE ratio is derived as:

$$R_{j}=\frac{p(x_{h},y_{h})}{p(x_{j},y_{j})}.\frac{1}{t}=\frac{qy_{h}-px_{h}}{qy_{j}-px_{j}}.k,\;\gamma_{j,h}\leq k\leq \rho_{j,h}.$$ (7)

In the above expression, *k* is the radial scale factor obtained from comparing the input and output vectors of *DMU*_*j*_ and the reference unit *h*. Taking *γ*_*j*,*h*_≤*k*≤*ρ*_*j*,*h*_ into account, *k* is only defined where *γ*_*j*,*h*_≤*ρ*_*j*,*h*_; otherwise, *k* is considered to be equivalent to zero.

To expand *R*_*j*_ into an efficiency criterion, the concept of OSSP is defined as a scale that maximizes the average radial profit. The following definition is presented as an extension of the OSS description provided by Cesaroni and Giovannola [2] for the profit framework:

**Definition 1.** For any given *DMU*_*j*_, an OSSP is a production possibility that maximizes *R*_*j*_.

According to Definition 1, an OSSP is an optimal solution to the following program:

$$\begin{array}{l} \max R_{j}=\frac{qy_{h}-px_{h}}{qy_{j}-px_{j}}.k\\ s.t.\;\gamma_{j,h}\leq k\leq \rho_{j,h},\\ \;(x_{h},y_{h})\in T. \end{array}$$ (8)

Taking *T* = *T*^*FDH*^ into consideration, model (8) transformed into the following model:

$$\begin{array}{l} max\;R_{j}=\frac{qy_{h}-px_{h}}{qy_{j}-px_{j}}.k\\ s.t.\;\left\{ \begin{array}{l} k=\rho_{j,h}\;if\;\gamma_{j,h}\leq \rho_{j,h},\\ k=0\;o.w., \end{array}\right.\\ \;(x_{h},y_{h})\in T^{FDH}. \end{array}$$ (9)

Model (9) is always feasible because (*qy*_*h*_−*px*_*h*_).k never becomes infinite and *x*_*h*_ = *x*_*j*_ and *y*_*h*_ = *y*_*j*_ is a feasible solution to this Model. To illustrate, *k* = *ρ*_*j*,*h*_ and (*qy*_*h*_−*px*_*h*_) are both numbers and the product of them will also be a number if *γ*_*j*,*h*_≤*ρ*_*j*,*h*_, and if *γ*_*j*,*h*_>*ρ*_*j*,*h*_, then *R*_*j*_ = 0. Also, since each unit can be treated as its reference, then $1\leq R_{j}^{*}<\infty$.

At this moment, we can define the APE measure as follows:

**Definition 2.** The APE measure of a *DMU* is the maximum value given by the solution of model (9). So,

$$APE_{j}=\underset{\left(x_{h},y_{h}\right)}{\max}R_{j}=\underset{\left(x_{h},y_{h}\right)}{\max}\frac{qy_{h}-px_{h}}{qy_{j}-px_{j}}.k.$$ (10)

Now model (8) *T*^*VRS*^ is considered. The following proposition shows the relationship between the value of APE in *T*^*VRS*^ and profit efficiency score under the CRS assumption

**Proposition 1.** The APE measure of a DMU under VRS is equivalent to its profit efficiency measure in constant returns to scale model.

**Proof.** By replacing *T*^*FDH*^ with *T*^*VRS*^in model (8), we have:

$$\begin{array}{l} R_{j}^{*}=\max\frac{qy_{h}-px_{h}}{qy_{j}-px_{j}}.\frac{1}{t}\\ s.t.\;ty_{j}\leq y_{h},\\ \;tx_{j}\geq x_{h},\\ \;(x_{h},y_{h})\in T^{VRS}. \end{array}$$

Based on the definition of *T*^*VRS*^, we have:

$$\begin{array}{c} \sum_{j=1}^{n}x_{ij}\lambda_{j}\leq x_{h},\\ \sum_{j=1}^{n}y_{rj}\lambda_{j}\geq y_{h}\\ \sum_{j=1}^{n}y_{rj}\lambda_{j}=1. \end{array},$$

By dividing the two sides of the constraints by *t*>0,*t*≠1, the following model is obtained

$$\begin{array}{l} R_{j}^{*}=\max\frac{q\frac{y_{h}}{t}-p\frac{x_{h}}{t}}{qy_{j}-px_{j}}\\ s.t.\;y_{j}\leq \frac{y_{h}}{t},\\ \;x_{j}\geq \frac{x_{h}}{t},\\ \;\sum_{j=1}^{n}x_{j}\frac{\lambda_{j}}{t}\leq \frac{x_{h}}{t},\\ \;\sum_{j=1}^{n}y_{j}\frac{\lambda_{j}}{t}\geq \frac{y_{h}}{t},\\ \;\sum_{j=1}^{n}\frac{\lambda_{j}}{t}=\frac{1}{t}. \end{array}$$

Using the changes of variables, i.e. $\frac{1}{t}=s^{*},\frac{\lambda_{j}}{t}=\lambda_{j}^{*},\frac{y_{h}}{t}=y^{*}$ $\frac{x_{h}}{t}=x^{*}$, the following linear model is achieved:

$$\begin{array}{c} R_{j}^{*}=max\frac{qy^{*}-px^{*}}{qy_{j}-px_{j}}\\ s.t.\;y_{j}\leq y^{*},\\ x_{j}\geq x^{*},\\ \sum_{j=1}^{n}x_{j}\lambda_{j}^{*}\leq x^{*},\\ \sum_{j=1}^{n}y_{j}\lambda_{j}^{*}\geq y^{*},\\ \sum_{j=1}^{n}\lambda_{j}^{*}=s^{*},\\ \lambda_{j}^{*},s^{*}\geq 0. \end{array}$$

Proposition 1 shows that an OSSP under VRS is a combination of inputs and outputs that maximizes the total profit under CRS.

According to Proposition 1 and the points stated concerning under *T*^*FDH*^, we can calculate the APE and the OSSP of a *DMU* in convex and non-convex technologies in the following way:

#### Case I: APE and OSSP in the non-convex FDH technology

The value of the objective function of model (9) for each unit under evaluation is calculated. The unit that provides the highest value of the objective function *DMU*_*j*_ is considered as *OSSP*_*j*_ and the estimated value is treated as *APE*_*j*_.

#### Case II: APE and OSSP in the convex VRS technology

Solve the following profit model and calculate the values of $\lambda_{j}^{*}$, $x_{ih}^{*}$ and $y_{rh}^{*}$:

$$\begin{array}{l} \max\sum_{r=1}^{s}q_{r}y_{rh}-\sum_{i=1}^{m}p_{i}x_{ih}\\ s.t.\;y_{ro}\leq y_{rh},\;r=1,2,\ldots ,s,\\ \;x_{io}\geq x_{ih},\;i=1,2,\ldots ,m,\\ \;\sum_{j=1}^{n}x_{ij}\lambda_{j}\leq x_{ih},\;i=1,2,\ldots ,m,\\ \;\sum_{j=1}^{n}y_{rj}\lambda_{j}\geq y_{rh},\;r=1,2,\ldots ,s,\\ \;\lambda_{j}\geq 0,x_{ih},y_{rh}\geq 0 \end{array}$$ (11)

Considering the results found by solving model (11), the value of *APE*_*o*_ can be obtained from the next statement:

$$APE_{o}=\frac{\sum_{r=1}^{s}q_{r}y_{rh}^{*}-\sum_{i=1}^{m}p_{i}x_{ih}^{*}}{\sum_{r=1}^{s}q_{r}y_{ro}-\sum_{i=1}^{m}p_{i}x_{io}}$$ (12)

Also, the convex combination of the reference points *DMU*_*o*_ under CRS with weights $\lambda_{j}^{*}/\sum_{j=1}^{n}\lambda_{j}^{*}$ is considered *OSSP*_*o*_ under VRS.

It can be easily shown that *APE*_*o*_ = 1 is a necessary and sufficient condition for contemplating *DMU*_*o*_ an *OSSP*.

Because of the presence of random measures in many real applications, in the next section, the approaches rendered herein are generalized to determine APE and OSSP under uncertainty.

### Determining APE and OSSP in the presence of stochastic data

Economic and political factors, the impact of unexpected government decisions, currency fluctuations, and environmental and agricultural factors beyond the control of the manager lead to perceiving uncertain data in many real-world applications. Thus, it is necessary to utilize stochastic programming methods to analyze the performance of entities in which inputs, outputs, or both of them may be random measures. As known, what is important in using stochastic programming models is that the probability distribution function of the data must be specific or measurable. The accessibility of the distribution and dispersion of data makes the prediction of the events more possible.

Chance-constrained DEA approaches such as the ones provided by Olesen and Petersen [4], Cooper et al. [39, 40] are among the beneficial methods to solve programming problems with random inputs and outputs. In the following, the chance-constrained DEA approach is proposed to estimate APE and OSSP in the presence of random inputs and outputs. Accordingly, we assume *DMU*_*j*_ uses *m* random inputs ${\tilde{x}}_{j}=({\tilde{x}}_{1j},\ldots ,{\tilde{x}}_{mj})$ to produce *s* random outputs ${\tilde{y}}_{j}=({\tilde{y}}_{1j},\ldots ,{\tilde{y}}_{sj})$. The vectors *x*_*j*_ = (*x*_1*j*_,…,*x*_*mj*_) *y*_*j*_ = (*y*_1*j*_,…,*y*_*sj*_) are also considered as the average vectors corresponding to the inputs and outputs, which are positive. In this case, the next model with stochastic constraints is presented to determine *APE*_*o*_:

$$\begin{array}{l} \max\;\sum_{r=1}^{s}q_{r}y_{rh}-\sum_{i=1}^{m}p_{i}x_{ih}\\ s.t.\;P(y_{ro}\leq y_{rh})\geq 1-\alpha ,\;r=1,2,\ldots ,s,\\ \;P(x_{io}\geq x_{ih})\geq 1-\alpha ,\;i=1,2,\ldots ,m,\\ \;P(\sum_{j=1}^{n}x_{ij}\lambda_{j}\leq x_{ih})\geq 1-\alpha ,\;i=1,2,\ldots ,m,\\ \;P(\sum_{j=1}^{n}y_{rj}\lambda_{j}\geq y_{rh})\geq 1-\alpha ,\;r=1,2,\ldots ,s,\\ \;\lambda_{j}\geq 0,x_{ih},y_{rh}\geq 0, \end{array}$$ (13)

in which *α* is a predefined number between zero and one and *P* indicates the probability.

Following Cooper et al. [40–42], we deem inputs and outputs in the form of random variables with multivariate normal distribution and identified parameters. To assess the maximum profit of the unit under investigation, model (13) should be converted into the deterministic form. For this purpose, the presented method by Cooper et al. [40, 41] is applied. The details of the procedure are presented in the following:

Consider the constraint $P(\sum_{j=1}^{n}{\tilde{x}}_{ij}\lambda_{j}\leq x_{ih})\geq 1-\alpha$. By defining variables $s_{i}^{-}\geq 0$, we have:

$$P(\sum_{j=1}^{n}{\tilde{x}}_{ij}\lambda_{j}-x_{ih}\leq -s_{i}^{-})=1-\alpha$$ (14)

and we put:

$${\tilde{d}}_{i}=\sum_{j=1}^{n}{\tilde{x}}_{ij}\lambda_{j}-x_{ih}.$$

Given that each linear combination of normally distributed random variables has also a normal distribution, we have:

$${\tilde{d}}_{i}\approx N(d_{i},\sigma_{i}^{2}(\lambda ))$$

where

$$\begin{array}{l} d_{i}=E(\sum_{j=1}^{n}{\tilde{x}}_{ij}\lambda_{j}-x_{ih})=\sum_{j=1}^{n}x_{ij}\lambda_{j}-x_{ih},\\ \sigma_{i}^{2}(\lambda )=\mathrm{var}({\tilde{d}}_{i})=\mathrm{var}(\sum_{j=1}^{n}{\tilde{x}}_{ij}\lambda_{j}-x_{ih})=\mathrm{var}(\sum_{j=1}^{n}{\tilde{x}}_{ij}\lambda_{j})=\sum_{j=1}^{n}\sum_{k=1}^{n}\lambda_{j}\lambda_{k}Cov({\tilde{x}}_{ij},{\tilde{x}}_{ik}). \end{array}$$

Considering the random variable ${\tilde{d}}_{i}=\sum_{j=1}^{n}{\tilde{x}}_{ij}\lambda_{j}-x_{ih}$ in the constraint (14), we have:

$$P({\tilde{d}}_{i}\leq -s_{i}^{-})=1-\alpha ,$$

or

$$P(\frac{{\tilde{d}}_{i}-d_{i}}{\sigma_{i}(\lambda )}\leq \frac{-s_{i}^{-}-d_{i}}{\sigma_{i}(\lambda )})=1-\alpha .$$

On the other hand, by replacing ${\tilde{Z}}_{i}=\frac{{\tilde{d}}_{i}-d_{i}}{\sigma_{i}(\lambda )}$ and considering ${\tilde{Z}}_{i}$ the variable with the standard normal distribution, we have:

$$\begin{array}{l} P({\tilde{Z}}_{i}\leq \frac{-s_{i}^{-}-d_{i}}{\sigma_{i}(\lambda )})=1-\alpha \\ P({\tilde{Z}}_{i}\geq \frac{s_{i}^{-}+d_{i}}{\sigma_{i}(\lambda )})=\alpha \\ \varphi (\frac{s_{i}^{-}+d_{i}}{\sigma_{i}(\lambda )})=\alpha \end{array}$$

In the above relationship, *φ* is the standard normal cumulative distribution function and *φ*^−1^(*α*) is its inverse (called fractile function). So we have:

$$\frac{s_{i}^{-}+d_{i}}{\sigma_{i}(\lambda )}=\varphi^{-1}(\alpha )\to s_{i}^{-}+d_{i}-\varphi^{-1}(\alpha ).\sigma_{i}(\lambda )=0.$$

Therefore, the deterministic form of constraint (14) will be as follows:

$$\begin{array}{l} \sum_{j=1}^{n}x_{ij}\lambda_{j}-\varphi^{-1}(\alpha )\sigma_{i}(\lambda )\leq x_{ih},\\ \sigma_{i}^{2}(\lambda )=\sum_{j=1}^{n}\sum_{k=1}^{n}\lambda_{j}\lambda_{k}Cov({\tilde{x}}_{ij},{\tilde{x}}_{ik}). \end{array}$$

Similarly, the constraint $P(\sum_{j=1}^{n}{\tilde{y}}_{rj}\lambda_{j}\geq y_{rh})\geq 1-\alpha$ is transformed into the following deterministic framework:

$$\begin{array}{l} \sum_{j=1}^{n}y_{rj}\lambda_{j}+\varphi^{-1}(\alpha )\delta_{r}(\lambda )\geq y_{rh},\\ \delta_{r}^{2}(\lambda )=\sum_{j=1}^{n}\sum_{k=1}^{n}\lambda_{j}\lambda_{k}Cov({\tilde{y}}_{rj},{\tilde{y}}_{rk}). \end{array}$$

Also, the next can be examined for the constraint $P({\tilde{x}}_{io}\geq x_{ih})\geq 1-\alpha$:

$$P(x_{ih}-{\tilde{x}}_{io}\leq -s_{i}^{-})=1-\alpha$$

By considering variables ${\tilde{k}}_{io}=x_{ih}-{\tilde{x}}_{io}$, we know ${\tilde{k}}_{io}\approx N(k_{io},\sigma_{io}^{2})$ where

$$\begin{array}{l} k_{io}=E(x_{ih}-{\tilde{x}}_{io})=x_{ih}-x_{io},\\ \sigma_{io}^{2}=\mathrm{var}({\tilde{k}}_{io})=\mathrm{var}(x_{ih}-{\tilde{x}}_{io})=\mathrm{var}({\tilde{x}}_{io}). \end{array}$$

Therefore, this constraint is written as deterministic in the following way:

$$x_{io}\geq x_{ih}-\varphi^{-1}(\alpha )\sigma_{io}.$$

Similarly for the constraints $P({\tilde{y}}_{ro}\leq y_{rh})\geq 1-\alpha$, we have:

$$y_{ro}\leq y_{rh}+\varphi^{-1}(\alpha )\delta_{ro},\;\delta_{ro}^{2}=\mathrm{var}({\tilde{y}}_{ro}).$$

Therefore, the chance-constrained profit model (13) is converted to the following quadratic problem:

$$\begin{array}{l} \max\sum_{r=1}^{s}q_{r}y_{rh}-\sum_{i=1}^{m}p_{i}x_{ih}\\ s.t.\;y_{ro}\leq y_{rh}+\varphi^{-1}(\alpha )\delta_{ro},\;r=1,2,\ldots ,s,\\ \;x_{io}\geq x_{ih}-\varphi^{-1}(\alpha )\sigma_{io},\;i=1,2,\ldots ,m,\\ \;\sum_{j=1}^{n}x_{ij}\lambda_{j}-\varphi^{-1}(\alpha )\sigma_{i}(\lambda )\leq x_{ih},\;i=1,2,\ldots ,m,\\ \;\sum_{j=1}^{n}y_{rj}\lambda_{j}+\varphi^{-1}(\alpha )\delta_{r}(\lambda )\geq y_{rh},\;r=1,2,\ldots ,s,\\ \;\lambda_{j}\geq 0,x_{ih},y_{rh}\geq 0. \end{array}$$ (15)

Model (15), *φ*(*α*) is the standard normal cumulative distribution function and *φ*^−1^(*α*) is its inverse function. We also have:

$$\begin{array}{l} \delta_{r}^{2}(\lambda )=\sum_{j=1}^{n}\sum_{k=1}^{n}\lambda_{j}\lambda_{k}Cov({\tilde{y}}_{rj},{\tilde{y}}_{rk}),\\ \sigma_{i}^{2}(\lambda )=\sum_{j=1}^{n}\sum_{k=1}^{n}\lambda_{j}\lambda_{k}Cov({\tilde{x}}_{ij},{\tilde{x}}_{ik}),\\ \delta_{ro}^{2}=Var({\tilde{y}}_{ro}),\\ \sigma_{io}^{2}=Var({\tilde{x}}_{io}), \end{array}$$ (16)

Model (15) is a quadratic nonlinear model due to the existence of quadratic constraints described in the expressions (16). However, if we consider input and output variables independent through the error structure provided by Huang and Li [43], a linear model for calculating APE can be provided. For more details, assume the inputs and outputs of the *j* th unit are as follows:

$$\begin{array}{l} {\tilde{x}}_{ij}=x_{ij}+a_{ij}{\tilde{\varepsilon }}_{ij},\;i=1,2,\ldots ,m,\\ {\tilde{y}}_{rj}=y_{rj}+b_{rj}{\tilde{\zeta }}_{rj},\;r=1,2,\ldots ,s, \end{array}$$ (17)

in which values *a*_*ij*_ and *b*_*rj*_ are non-negative and random variables ${\tilde{\varepsilon }}_{ij}$ and ${\tilde{\zeta }}_{rj}$ are normal so that ${\tilde{\varepsilon }}_{ij}\approx N(0,{\bar{\sigma }}^{2}),{\tilde{\zeta }}_{rj}\approx N(0,{\bar{\delta }}^{2})$. In these cases, ${\tilde{\varepsilon }}_{ij}$ and ${\tilde{\zeta }}_{rj}$ are the errors of the random input and output variables. Based on relationships (17) we have:

$$\begin{array}{l} {\tilde{x}}_{ij}\approx N(x_{ij},{\bar{\sigma }}^{2}a_{ij}^{2}),\\ {\tilde{y}}_{rj}\approx N(y_{rj},{\bar{\delta }}^{2}b_{rj}^{2}). \end{array}$$ (18)

By assuming the independence of the input and output variables of distinct DMUs, $Cov({\tilde{y}}_{rj},{\tilde{y}}_{rk})=0,Cov({\tilde{x}}_{ij},{\tilde{x}}_{ik})=0$. It can also be supposed that ${\tilde{\varepsilon }}_{ij}={\tilde{\varepsilon }}_{i}$ and ${\tilde{\zeta }}_{rj}={\tilde{\zeta }}_{r}$. Taking these suppositions, model (13) can be transformed into the deterministic structure as follows:

Consider the constraints $P(\sum_{j=1}^{n}{\tilde{x}}_{ij}\lambda_{j}\leq x_{ih})\geq 1-\alpha ,(i=1,\ldots ,m)$. By introducing ${\tilde{d}}_{i}=\sum_{j=1}^{n}{\tilde{x}}_{ij}\lambda_{j}-x_{ih}$, the properties of the normal distribution function, the error structure, and the above assumptions, we have:

$${\tilde{d}}_{i}=\sum_{j=1}^{n}{\tilde{x}}_{ij}\lambda_{j}-x_{ih}=(\sum_{j=1}^{n}x_{ij}\lambda_{j}+\varepsilon_{i}\sum_{j=1}^{n}a_{ij}\lambda_{j})-x_{ih}=(\sum_{j=1}^{n}x_{ij}\lambda_{j}-x_{ih})+\varepsilon_{i}\sum_{j=1}^{n}a_{ij}\lambda_{j},$$

where

$${\tilde{d}}_{i}\approx N(\sum_{j=1}^{n}x_{ij}\lambda_{j}-x_{ih},{\bar{\sigma }}^{2}{\left(\sum_{j=1}^{n}a_{ij}\lambda_{j}\right)}^{2}).$$

Therefore, the aforementioned constraints transform into the following deterministic framework:

$$\sum_{j=1}^{n}x_{ij}\lambda_{j}-\varphi^{-1}(\alpha )\bar{\sigma }|\sum_{j=1}^{n}a_{ij}\lambda_{j}|\leq x_{ih}.$$

Considering the positive values *a*_*ij*_, the above-mentioned constraints will be linear:

$$\sum_{j=1}^{n}x_{ij}\lambda_{j}-\varphi^{-1}(\alpha )\bar{\sigma }(\sum_{j=1}^{n}a_{ij}\lambda_{j})\leq x_{ih}.$$

In the same vein, the constraints $P(\sum_{j=1}^{n}{\tilde{y}}_{rj}\lambda_{j}\geq y_{rh})\geq 1-\alpha ,(r=1,\ldots ,s)$ will be linear as follows:

$$\sum_{j=1}^{n}y_{rj}\lambda_{j}+\varphi^{-1}(\alpha )\bar{\sigma }(\sum_{j=1}^{n}b_{rj}\lambda_{j})\geq y_{rh}.$$

We also define variables ${\tilde{k}}_{io}=x_{ih}-{\tilde{x}}_{io}$ for constraints $P({\tilde{x}}_{io}\geq x_{ih})\geq 1-\alpha$ and have:

$${\tilde{k}}_{io}=x_{ih}-{\tilde{x}}_{io}=(x_{ih}-x_{io})+\varepsilon_{i}(-a_{io}).$$

Therefore, the deterministic equivalent of these constraints will be as follows:

$$x_{io}\geq x_{ih}-\varphi^{-1}(\alpha )\bar{\sigma }|-a_{io}|.$$

Due to the positive values *a*_*io*_, the linear form of these constraints can be written as follows:

$$x_{io}\geq x_{ih}-\varphi^{-1}(\alpha )\bar{\sigma }a_{io}.$$

By taking the analogous process for constraints $P({\tilde{y}}_{ro}\leq y_{rh})\geq 1-\alpha ,(r=1,\ldots ,s)$, the stochastic model for calculating *APE*_*o*_ can be converted into the following linear problem:

$$\begin{array}{l} \max\;\sum_{r=1}^{s}q_{r}y_{rh}-\sum_{i=1}^{m}p_{i}x_{ih}\\ s.t.\;y_{ro}\leq y_{rh}+\varphi^{-1}(\alpha )\bar{\sigma }b_{ro},\;r=1,2,\ldots ,s,\\ \;x_{io}\geq x_{ih}-\varphi^{-1}(\alpha )\bar{\sigma }a_{io},\;i=1,2,\ldots ,m,\\ \;\sum_{j=1}^{n}x_{ij}\lambda_{j}-\varphi^{-1}(\alpha )\bar{\sigma }(\sum_{j=1}^{n}a_{ij}\lambda_{j})\leq x_{ih},\;i=1,2,\ldots ,m,\\ \;\sum_{j=1}^{n}y_{rj}\lambda_{j}+\varphi^{-1}(\alpha )\bar{\sigma }(\sum_{j=1}^{n}b_{rj}\lambda_{j})\geq y_{rh},\;r=1,2,\ldots ,s,\\ \;\lambda_{j}\geq 0,x_{ih},y_{rh}\geq 0. \end{array}$$ (19)

After obtaining the optimal values $x_{ih}^{*}$ and $y_{rh}^{*}$ along with computing model (19), the value of *APE*_*o*_ results from the following expression:

$$APE_{o}=\frac{\sum_{r=1}^{s}q_{r}y_{rh}^{*}-\sum_{i=1}^{m}p_{i}x_{ih}^{*}}{\sum_{r=1}^{s}q_{r}{\tilde{y}}_{ro}-\sum_{i=1}^{m}p_{i}{\tilde{x}}_{io}}=\frac{\sum_{r=1}^{s}q_{r}y_{rh}^{*}-\sum_{i=1}^{m}p_{i}x_{ih}^{*}}{\left(\sum_{r=1}^{s}q_{r}y_{ro}-\sum_{i=1}^{m}p_{i}x_{io})+\varphi^{-1}(\alpha )(\sum_{r=1}^{s}q_{r}b_{ro}-\sum_{i=1}^{m}p_{i}a_{io}\right)}.$$ (20)

The *APE*_*o*_ value in the expression (20) is gained as the ratio of the optimal value of the objective function of the model (19) to the random value of the profit of the unit under evaluation at the level *α*. Models (14) and (19) for the values *α*≥0.5 are always feasible. The details of this claim are given in Proposition 2.

**Proposition 2.** If *α*≥0.5, models (14) and (19) will be always feasible.

**Proof.** We consider model (14) and show it is feasible for *α*≥0.5. For model (19), it can also be proved similarly.

Suppose *λ*_*j*_ = 0(*j*≠*o*),*λ*_*o*_ = 1. In this case, the constraints of the model (14) will be as follows:

$$\begin{array}{l} y_{ro}-\varphi^{-1}(\alpha )\delta_{ro}\leq y_{rh},\\ x_{io}+\varphi^{-1}(\alpha )\sigma_{io}\geq x_{ih},\\ x_{io}-\varphi^{-1}(\alpha )\sigma_{i}(\lambda )\leq x_{ih},\\ y_{ro}+\varphi^{-1}(\alpha )\delta_{r}(\lambda )\geq y_{rh}. \end{array}$$

If *α*≥0.5, then *φ*^−1^(*α*)≥0. So, $y_{rh}=y_{ro},x_{ih}=x_{io},\lambda_{j}=0\;(j\neq o),\lambda_{o}=1$ is a solution for model (14). Therefore, the proof is complete.

It can be easily observed that if *α*<0.5, model (14) is not always feasible.

Therefore, if the inputs and outputs of DMUs are random and the production possibility set is under VRS, model (19) should be computed to calculate *APE*_*o*_ at the level *α*≥0.5, and then the value *APE*_*o*_ can be achieved using the expression (20). Given the constraints of the model (19), if *α*≥0.5, none of the units under evaluation are on the profit efficiency frontier, so the *APE* values of the units are not equal to one and there will be no OSSP. Containing data errors increases the production possibility set and naturally no unit is on the new frontier. In this case, to calculate *OSSP*_*o*_ under VRS, the convex combination of the reference points *DMU*_*o*_ under CRS with weights $\lambda_{j}^{*}/\sum_{j=1}^{n}\lambda_{j}^{*}$ can be used in which $\lambda_{j}^{*}(j=1,2,\ldots ,n)$ are the optimal values resulted from model (19). It should be noted that under CRS, each reference point of model (19) can be considered as the OSSP.

## Practical example

In this section, the proposed model is applied to evaluate the performance of ten postal regions in Iran. The performance analysis of postal regions is notable in economic development. As Çakır et al. [44] mentioned, there are not many studies that analyze the performance of postal areas. We, therefore, consider the information of ten postal areas in Iran according to data availability. For this purpose, the data related to postal areas with one input *x*_1_ and three outputs, *y*_1_,*y*_2_ and *y*_3_ have been extracted as follows:

*x*_1_: Personal and non-personal expenses

*y*_1_: Number of regular, express, custom, and special shipments accepted

*y*_2_: Number of electronic service shipments

*y*_3_: Sale services

These measures are taken due to the importance, purpose, and consultation with managers. The data were collected for these postal regions during a period of 36 months from 2016 to 2018 with the management reporting system of the Post Company of the Islamic Republic of Iran and in cooperation with the Program and Budget Center of the National Post Company of the Islamic Republic of Iran. The mean of data is given in Table 1. In addition, the values of standard deviation in these three years are calculated and listed in Table 2. Notice that Parhizkar et al. [21] conducted their study on 12 postal areas, whereas this study has similar data from 9 entities.

**Table 1:** Mean of data for 2016–2018.

Postal areas	*x*_1_	*y*_1_	*y*_2_	*y*_3_
Esfahan	650	768889	105536	348754
Zanjan	162	297421	20096	83374
Ghazvin	194	389839	27646	128443
Gilan	599	446094	259354	68705
Mazandaran	539	767261	552835	272790
Alborz	420	731567	69860	150266
District 11 of Tehran	368	3861296	57364	262535
District 13 of Tehran	412	975355	413537	770283
District 14 of Tehran	501	1849588	45607	266943
District 15 of Tehran	292	1869858	342620	587337

**Table 2:** The standard deviation of data for 2016–2018.

Postal areas	*x*_1_	*y*_1_	*y*_2_	*y*_3_
Esfahan	35	369441	55192	158670
Zanjan	3.5	200151	30385	118220
Ghazvin	4.5	248655	14192	166474
Gilan	33	261066	250283	17754
Mazandaran	22	367447	305195	303553
Alborz	6.5	222084	26672	125530
District 11 of Tehran	23	2513351	172249	179445
District 13 of Tehran	18.5	170965	395844	530308
District 14 of Tehran	25	389274	31249	530308
District 15 of Tehran	28	2976924	400970	672770

Due to the same service costs for all postal areas, the input and output prices of the units are deemed the same and are calculated by averaging the prices of these three years as follows:

*p*: Average personnel and non-personnel costs of units per personnel

*q*_1_: Average revenue from accepting regular—special shipments per traffic

*q*_2_: Average revenue resulted from accepting electronic service shipments per traffic

*q*_3_: Average revenue from sale services per traffic

Note that due to the lack of significant price changes during the three years, the above-mentioned prices are assumed as deterministic for these years.

First, to calculate the APE of the units in the deterministic state and in the FDH space, we calculate model (9) for all the units. In this case, we consider the average values of the inputs and outputs as the deterministic data of the units. Implementing the counting algorithm for units leads to calculating the results of APE of units. These results are given in the second column of Table 3.

**Table 3:** The APE measures of postal areas.

Postal areas	*α* = 0.5	*α* = 0.75	*α* = 0.84	FDH
Esfahan	-7.81	-24.6	-48.5	-7.81
Zanjan	-18.3	22.66	16.90	-18.3
Ghazvin	-42.7	16.43	13.96	-42.7
Gilan	-5.21	-15.8	-27.4	-5.21
Mazandaran	-84.8	18.9	16.75	-84.8
Alborz	-14.1	-126	207.8	-14.1
District 11 of Tehran	1.00	1.20	1.39	1.00
District 13 of Tehran	4.01	5.87	6.54	4.01
District 14 of Tehran	6.03	8.25	9.83	6.03
District 15 of Tehran	1.00	1.16	1.24	1.00

As can be seen in the Table 3, the first to sixth units have negative APE. This means that the mentioned units have been unprofitable units in the three years under review. The APE of 8th and 9th units was positive, but more than one. This means that these two units, although being profitable, are not OSS. Finally, the 7th and 10th units having an APE equal to one, are OSSP units under FDH technology.

Now, we calculate the APE of postal areas in convex and stochastic environment. For this purpose, considering that the input and output values of the units are stochastic with the mean and standard deviation presented in Tables 1 and 2 and assuming that errors in the inputs and outputs of all units are the same along with lack of correlation between the inputs and outputs, the model (19) has been used and the results of this model for three values of *α* are shown in the Table 3.

As can be seen in Table 3, if we consider *α* = 0.5 and put its corresponding value namely *φ*^−1^(*α*) = 0 in model (19), the results of APE for units are the same as the results of the third column of Table 3. In fact, the calculation of APE in the case of *α* = 0.5 is the same as the calculation of the APE of units in the convex case and with the definition of average data for the units. Therefore, the random model (19) will be the same as the deterministic model (11) in the case of *α* = 0.5. Therefore, as can be seen in the Table 3, these values are exactly the same as the calculated values for APE in FDH model, which can be observed in the second column. In fact, this similarity of efficiency is due to the equivalence that was shown in theorem 1 and its result can be observed in the practical example.

For *α* = 0.75, the APE values estimated through model (19) and statement (20) are shown in the third column of Table 3. As shown in this column, Zanjan, Ghazvin, and Mazandaran have positive APE values and which means that considering *α* = 0.75, these units will be profitable. Meanwhile, these units at the level *α* = 0.5 were unprofitable. Also, at the level *α* = 0.75, none of the units are OSSP, because none of them get the APE value equals to one. Note that if the ranking of units at this level is examined, the tenth unit with the lowest absolute value of APE compared to others will be selected as the best one. However, this unit is not OSSP and therefore can attempt to reach the OSSP level.

The results of APE of the units for *α* = 0.84 are also presented in the fourth column of Table 3. At this level, Alborz is introduced as a profitable unit and similar to the level *α* = 0.75, none of these units are OSSP. Nevertheless, district 15 of Tehran can be selected as the most efficient unit under evaluation again.

To determine the OSSP of any area, as long as *α* = 0.5, the convex combination of the reference points of each area with weights $\lambda_{j}^{*}/\sum_{j=1}^{n}\lambda_{j}^{*}$ is calculated and listed in Table 4.

**Table 4:** The OSSP of postal areas at confidence level *α* = 0.5.

Postal areas	*x*_1_	*y*_1_	*y*_2_	*y*_3_
Esfahan	367	3845332	59651	265139
Zanjan	368	3861296	57364	262535
Ghazvin	368	3861296	57364	262535
Gilan	344	3223034	148789	366635
Mazandaran	301	2106309	308750	548772
Alborz	367	3835581	61047	266729
District 11 of Tehran	368	3861296	57364	262535
District 13 of Tehran	299	2046476	317321	558531
District 14 of Tehran	368	3861296	57364	262535
District 15 of Tehran	292	1869858	342620	587337

As can be seen in Table 4, for the seventh and tenth areas which are OSSP at this level α, the determined OSSP is the same area under evaluation, but for non-OSSP areas like the second area, OSSP is the seventh unit. For the first area, the combination of seventh and tenth units is determined as OSSP.

Considering *α* = 0.75, the only OSSP point for all the units is the combination point $\left(x_{1}=273,\;y_{1}=3894166,\;y_{2}=615279,\;y_{3}=1044821\right)$, because the only unit affecting other units with *λ*_*j*_≠0 is the tenth unit; therefore, the values $\lambda_{j}^{*}/\sum_{j=1}^{n}\lambda_{j}^{*}$ are the same for all the units and a similar OSSP is obtained for all areas. This is because of the VRS assumption and the reference points of each unit can be considered as its OSSP without including this assumption, In this case, different OSSPs for areas are found.

In order to analyse the sensitivity of the OSSP with respect to the error term, we get the results for two confidence levels *α* = 0.3 and *α* = 0.1. The results are listed in Tables 5 and 6, respectively.

**Table 5:** The OSSP of postal areas at confidence level *α* = 0.3.

Postal areas	*x*_1_	*y*_1_	*y*_2_	*y*_3_
Esfahan	365	3845344	59659	265149
Zanjan	361	3861298	57364	262575
Ghazvin	361	3861299	57364	262555
Gilan	340	3223067	148789	366635
Mazandaran	300	2106311	308750	548772
Alborz	365	3835597	61047	266759
District 11 of Tehran	368	3861296	57364	262535
District 13 of Tehran	296	2046476	317333	558541
District 14 of Tehran	368	3861296	57364	262535
District 15 of Tehran	292	1869858	342620	587337

**Table 6:** The OSSP of postal areas at confidence level *α* = 0.1.

Postal areas	*x*_1_	*y*_1_	*y*_2_	*y*_3_
Esfahan	364	3845344	59659	265159
Zanjan	361	3861298	57364	262585
Ghazvin	361	3861299	57364	262555
Gilan	340	3223067	148789	366655
Mazandaran	300	2106332	308750	548772
Alborz	363	3835597	61047	266769
District 11 of Tehran	368	3861296	57364	262535
District 13 of Tehran	293	2046478	317333	558551
District 14 of Tehran	368	3861296	57364	262535
District 15 of Tehran	292	1869858	342620	587337

## Conclusion

In this paper, two new steps were presented regarding the evaluation of the profit performance of the units. In the first step, the concepts of ACE and OSS, which are presented in the DEA literature based on costs of inputs, were developed for profit, and the concepts of APE and OSSP which have the same characteristics as those presented for ACE and OSS for profit were defined. Equivalently, APE was presented as a valuable and more accurate measure of efficiency than profit efficiency in non-convex space, and then it was shown that the model presented for calculating APE in convex space and variable returns to scale is the same profit model under the constant returns to scale. Therefore, this model can be applied to calculate APE and OSSP in convex space.

In the second step, the APE model presented in the first step was developed in the stochastic environment so that this model can calculate the APE values of units with stochastic inputs and outputs. This model, which was a probabilistic model with deterministic objective function and probabilistic constraints, was transformed into a second-order deterministic model during a process. It was also shown that, assuming the variables are uncorrelated, the non-linear deterministic model can be converted into a linear model. Accordingly, if the input and output prices of the units are available, in the first step, calculating the APE and OSSP values of the units in the non-convex space will provide valuable and far more accurate evaluations than the profit efficiency evaluation of the units, and in the second step, using the proposed stochastic models will provide a more accurate and realistic evaluation of the units’ profit efficiency compared to the deterministic models presented for the units’ profit efficiency.

There are some limitations need to be noted: First, the availability of the inputs and outputs data are our major limitation. Second, we assumed that the random data follow normal distribution with known mean and variance. However, in real applications, this may be violated.

At the end, the following suggestions are provided for future studies:

- Presenting the Malmquist index of cost, income, and profit based on the distance functions of cost, income, and profit and developing this index in the stochastic case.
- Developing the proposed approach to situations that the fuzzy stochastic parameters are presented.

## References

[1] Charnes A., Cooper W. W., & Rhodes E. (1978). Measuring the efficiency of decision-making units. *European journal of operational research*, 2(6), 429–444.

[2] Cesaroni G., & Giovannola D. (2015). Average-cost efficiency and optimal scale sizes in non-parametric analysis. *European Journal of Operational Research*, 242(1), 121–133.

[3] Land K. C., Lovell C. K., & Thore S. (1993). Chance-constrained data envelopment analysis. *Managerial and decision economics*, 14(6), 541–554.

[4] Olesen O. B., & Petersen N. C. (1995). Chance-constrained efficiency evaluation. *Management Science*, 41(3), 442–457.

[5] Cooper W. W., Huang Z., Lelas V., Li S. X., & Olesen O. B. (1998). Chance-constrained programming formulations for stochastic characterizations of efficiency and dominance in DEA. *Journal of productivity analysis*, 9(1), 53–79.

[6] Ruggiero J. (2004). Data envelopment analysis with stochastic data. *Journal of the Operational Research Society*, 55(9), 1008–1012.

[7] Khanjani Shiraz R., Tavana M., & Fukuyama H. (2021). A joint chance-constrained data envelopment analysis model with random output data. *Operational Research*, 21(2), 1255–1277.

[8] Mehdizadeh S., Amirteimoori A., Charles V., Behzadi M. H., & Kordrostami S. (2021). Measuring the efficiency of two-stage network processes: A satisficing DEA approach. *Journal of the Operational Research Society*, 72(2), 354–366.

[9] Zhou Z., Sun W., Xiao H., Jin Q., & Liu W. (2021). Stochastic leader–follower DEA models for two-stage systems. *Journal of Management Science and Engineering*.

[10] Amirteimoori A., Khoshandam L., Kordrostami S., Jahani Sayyad Noveiri M., & Matin R. K. (2022). Performance analysis in a stochastic supply chain with reverse flows: a DEA-based approach. *IMA Journal of Management Mathematics*, 33(3), 433–456.

[11] Jahani Sayyad Noveiri M., Kordrostami S., & Amirteimoori A. (2021). Sustainability assessment and most productive scale size: a stochastic DEA approach with dual frontiers. *Environmental Modeling &* *Assessment*, 26, 723–735.

[12] Jahani Sayyad Noveiri M., Kordrostami S., & Karimi Omshi S. (2021). Average-Revenue Efficiency and Optimal Scale Sizes in Environmental Analysis. *Iranian Journal of Operations Research*, 12(2), 146–157.

[13] Khanjani Shiraz R. K., Hatami-Marbini A., Emrouznejad A., & Fukuyama H. (2020). Chance-constrained cost efficiency in data envelopment analysis model with random inputs and outputs. *Operational Research*, 20(3), 1863–1898.

[14] Farrell M. J. (1957). The measurement of productive efficiency. *Journal of the Royal Statistical Society*: *Series A (General)*, 120(3), 253–281.

[15] Frisch R. (1965). Theory of production. *Chicago*: *Rand McNally and Company*

[16] Baumol W. J., Panzar J. C., & Willig R. D. (1983). Contestable markets: An uprising in the theory of industry structure: Reply. *The American Economic Review*, 73(3), 491–496.

[17] Färe R., & Grosskopf S. (1985). A nonparametric cost approach to scale efficiency. *The Scandinavian Journal of Economics*, 594–604.

[18] Sueyoshi T. (1999). DEA duality on returns to scale (RTS) in production and cost analyses: an occurrence of multiple solutions and differences between production-based and cost-based RTS estimates. *Management Science*, 45(11), 1593–1608.

[19] Førsund F. R., & Hjalmarsson L. (2004). Are all scales optimal in DEA? Theory and empirical evidence. *Journal of Productivity Analysis*, 21(1), 25–48.

[20] Haghighatpisheh H., Kordrostami S., Amirteimoori A., & Lotfi F. H. (2022). Optimal scale sizes in input–output allocative data envelopment analysis models. *Annals of Operations Research*, 315(2), 1455–1476.

[21] Parhizkar Miyandehi L., Amirteimoori A., Kordrostami S., & Soufi M. (2022). Average revenue efficiency and optimal scale sizes in stochastic data envelopment analysis: A case study of post offices. Industrial Management Studies, 20(66), 73–110.

[22] Mitrović Simić J., Stević Ž., Zavadskas E. K., Bogdanović V., Subotić M., & Mardani A. (2020). A novel CRITIC-Fuzzy FUCOM-DEA-Fuzzy MARCOS model for safety evaluation of road sections based on geometric parameters of road. *Symmetry*, 12(12), 2006.

[23] Blagojević A., Vesković S., Kasalica S., Gojić A., & Allamani A. (2020). The application of the fuzzy AHP and DEA for measuring the efficiency of freight transport railway undertakings. *Operational Research in Engineering Sciences*: *Theory and Applications*, 3(2), 1–23.

[24] Akbarian D. (2020). Overall profit Malmquist productivity index under data uncertainty. *Financial Innovation*, 6(1), 1–20.

[25] Mitropoulos P., Zervopoulos P. D., & Mitropoulos I. (2020). Measuring performance in the presence of noisy data with targeted desirable levels: evidence from healthcare units. *Annals of Operations Research*, 294(1), 537–566.

[26] Agrawal N. (2021). An integrated benchmarking efficiency analysis of the Indian banking industry using data envelopment analysis. *International Journal of Process Management and Benchmarking*, 11(5), 671–692.

[27] Arsu T. (2021). Investigation into the efficiencies of European football clubs with bi-objective multi-criteria data envelopment analysis. *Decision Making*: *Applications in Management and Engineering*, 4(2), 106–125.

[28] Rasoulzadeh M., Edalatpanah S. A., Fallah M., & Najafi S. E. (2022). A multi-objective approach based on Markowitz and DEA cross-efficiency models for the intuitionistic fuzzy portfolio selection problem. *Decision Making*: *Applications in Management and Engineering*, 5(2), 241–259.

[29] Stević Ž., Miškić S., Vojinović D., Huskanović E., Stanković M., & Pamučar D. (2022). Development of a Model for Evaluating the Efficiency of Transport Companies: PCA–DEA–MCDM Model. *Axioms*, 11(3), 140.

[30] Fathi B. (2022). Measuring Super-Efficiency Using Stochastic Data Envelopment Analysis in Water and Wastewater Companies of Markazi Province. *Iran-Water Resources Research*, 17(4), 70–78.

[31] Kao C., & Liu S. T. (2019). Stochastic efficiency measures for production units with correlated data. *European Journal of Operational Research*, 273(1), 278–287.

[32] Piri M., Lotfi F. H., Rostamy-Malkhalifeh M., & Behzadi M. H. (2018). Evaluating decision-making units with stochastic data by the multiplier model in DEA. *Advances and Applications in Mathematical Sciences*, 17(5), 385–400.

[33] Gómez-Gallego J. C., Gómez-Gallego M., García-García J. F., & Faura-Martinez U. (2021). Evaluation of the Efficiency of European Health Systems Using Fuzzy Data Envelopment Analysis. *Healthcare (Basel*, *Switzerland)*, 9(10), 1270. doi: 10.3390/healthcare9101270 34682950 PMC8536069

[34] Ngo T., Tsui K.W.H. (2022). Estimating the confidence intervals for DEA efficiency scores of Asia-Pacific airlines. *Oper Res Int J* 22, 3411–3434.

[35] Chowdhury M. A. M., & Haron R. (2021). The efficiency of Islamic Banks in the Southeast Asia (SEA) region. *Future Business Journal*, 7(1), 1–16.

[36] Dar A. H., Mathur S. K., & Mishra S. (2021). The Efficiency of Indian Banks: A DEA, Malmquist and SFA Analysis with Bad Output. *Journal of Quantitative Economics*, 19(4), 653–701. doi: 10.1007/s40953-021-00247-x 34493912 PMC8413359

[37] Shah W. U. H., Hao G., Zhu N., Yasmeen R., Padda I. U. H., & Abdul Kamal M. (2022). A cross-country efficiency and productivity evaluation of commercial banks in South Asia: A meta-frontier and Malmquist productivity index approach. *Plos one*, 17(4), e0265349. doi: 10.1371/journal.pone.0265349 35385496 PMC8986021

[38] Jahani Sayyad Noveiri M., & Kordrostami S. (2022). Meta-Frontier Stochastic Cost and Revenue Efficiency Analysis: An Application to Bank Branches. *International Journal of Information Technology & Decision Making*, 1–25.

[39] Banker R. D., Charnes A., & Cooper W. W. (1984). Some models for estimating technical and scale inefficiencies in data envelopment analysis. *Management Science*, 30(9), 1078–1092.

[40] Cooper W. W., Deng H., Huang Z., & Li S. X. (2002). Chance-constrained programming approaches to technical efficiencies and inefficiencies in stochastic data envelopment analysis. *Journal of the Operational Research Society*, 53(12), 1347–1356.

[41] Cooper W. W., Deng H., Huang Z., & Li S. X. (2004). Chance-constrained programming approaches to congestion in stochastic data envelopment analysis. *European Journal of Operational Research*, 155(2), 487–501.

[42] Cooper W. W., Huang Z., & Li S. X. (1996). Satisficing DEA models under chance constraints. *Annals of operations research*, 66(4), 279–295.

[43] Huang Z., & Li S. X. (2001). Stochastic DEA models with different types of input-output disturbances. *Journal of Productivity Analysis*, 15(2), 95–113.

[44] Çakır S., Perçin S., & Min H. (2015). Evaluating the comparative efficiency of the postal services in OECD countries using context-dependent and measure-specific data envelopment analysis. *Benchmarking*: *An International Journal*, 22(5), 839–856.

